# Research on the relationship between physical activity, sleep quality, psychological resilience, and social adaptation among Chinese college students: A cross-sectional study

**DOI:** 10.3389/fpsyg.2023.1104897

**Published:** 2023-02-10

**Authors:** Yongbin Li, Kelei Guo

**Affiliations:** School of Physical Education and Health, Zhaoqing University, Zhaoqing, China

**Keywords:** physical activity, psychological resilience, social adaptation, sleep quality, college students

## Abstract

**Background:**

Sleep quality has become a frequent and prominent public health problem in Chinese universities, which seriously hinders the healthy development of college students and the improvement of the quality of higher education.

**Objective:**

The purpose of this study is to explore the relationship between physical activity and sleep quality among Chinese college students, and the mechanism of psychological resilience and social adaptation, and provide suggestions for improving the sleep quality among Chinese college students.

**Methods:**

From August to September 2022, a cross-sectional survey was conducted by using the convenience sampling method in Guangdong Province. 1,622 college students were investigated with *Physical Activity Scale*, *Pittsburgh Sleep Quality Index Scale* (PSQI), *Psychological Resilience Scale*, and *Social Adaptation Diagnostic Scale*, of which 893 were male and 729 were female. Use SPSS 23.0 and PROCESS plug-ins to analyze data.

**Results:**

(1) There was a significant negative correlation between physical activity and sleep quality (*r* = −0.237), and the direct path of physical activity to sleep quality was significant (β = −0.236, *t* = −9.888, *p* < 0.01); (2) physical activity can positively predict psychological resilience (β = 0.215, *t* = 8.823, *p* < 0.01) and social adaptation (β = 0.164, *t* = 7.773, *p* < 0.01); psychological resilience can negatively predict sleep quality (β = −0.337, *t* = −15.711, *p* < 0.01), positive prediction of social adaptation (β = 0.504, *t* = 23.961, *p* < 0.01); social adaptation can negatively predict sleep quality (β = −0.405, *t* = −18.558, *p* < 0.01); (3) psychological resilience and social adaptation play a significant mediating role between physical activity and sleep quality. The mediation effect consists of three paths: physical activity → psychological resilience → sleep quality (mediation effect value is −0.0723), physical activity → social adaptation → sleep quality (mediation effect value is −0.0662), and physical activity → psychological resilience → social adaptation → sleep quality (mediation effect value is −0.0438). (4) There is no gender difference in chain mediated effect.

**Conclusion:**

(1) Physical activity can significantly positively predict college students’ psychological resilience and social adaptation, and negatively predict sleep quality, which means that physical activity may help improve college students’ psychological resilience and social adaptation, and reduce sleep quality problems; (2) physical activity can not only directly affect the quality of sleep, but also indirectly affect the quality of sleep through the independent intermediary role of psychological resilience and social adaptation and the chain intermediary role of both. This further explains the reason why physical activity plays a role in college students’ sleep quality, which will help to provide some inspiration for colleges and universities to reduce college students’ sleep quality problems and formulate intervention plans.

## Introduction

Sleep quality is a composite measure of sleep duration, efficiency in falling asleep, and degree of deep sleep (Vargas et al., 2014). For college students, chronic sleep deprivation also means lower academic performance and an increased risk of burnout (Naderi et al., 2021), and seriously affects them academic life. Furthermore, if sleep problems persist for a long time and are not corrected in time, endocrine disruptions may turn into sleep disorders, leading to emotional problems such as anxiety and depression (Marvaldi et al., 2021), and even increasing the risk of suicidal ideation (Guo, 2021). Studies have shown that many Chinese college students are troubled by sleep problems (Tan and Liu, 2020; Fu and Zhang, 2021). Many scholars have studied this phenomenon and previous studies have shown that internal psychological factors such as mindfulness, stress, and anxiety can affect sleep quality (Xie et al., 2020; Zhang et al., 2021; Hu et al., 2022). At the same time, some scholars believe that external factors such as physical activity can also affect sleep quality (Ye et al., 2022). Therefore, this study considers both psychological and physical factors to reveal the potential mechanism of sleep quality, in order to provide empirical basis and theoretical framework for promoting college students’ sleep quality.

Nowadays, many studies believe that physical activity is an important factor affecting sleep quality, and a number of studies have shown that physical activity is negatively associated with sleep quality in college students (Ye et al., 2022), and the results of the meta-analysis further confirm that physical activity can increase and improve sleep quality in children and adolescents (Chen et al., 2022). The results of another study also showed that the proportion of students with psychopathological symptoms and sleep disorders was significantly lower in students with high levels of physical activity than in students with low levels of physical activity. This suggests that physical activity at a certain intensity can contribute positively to reducing the occurrence of psychological disorders and improving the quality of sleep. In a study by Passos et al. (2014), it was shown that scientific and effective exercise was effective in improving the quality of sleep of exercisers and also had a positive effect on reducing the incidence of depression in exercisers. Gerber et al. (2014) showed that college students who participated in vigorous physical activity had better sleep patterns, as well as more slow-wave sleep; conversely, those who participated in less intense physical activity had a higher proportion of light sleep. Although there have been many studies on the relationship between physical activity and sleep quality, there is still a lack of studies considering multiple factors at the same time. Recent studies have confirmed that the relationship between physical activity and sleep quality may be affected by many factors (Xie et al., 2020; Zhang et al., 2021; Hu et al., 2022).

In this paper, we investigate the influence of physical activity on sleep quality and the mediating role of psychological resilience and social adaptation, not only to enrich the theoretical scope of research on the factors influencing sleep quality in college students, but also to provide an empirical basis for improving and intervening in sleep quality in college students at the practical level. Thus, we propose the following hypotheses: H1. Physical activity can significantly negatively predict sleep quality among college students.

Psychological resilience may play an important role in the relationship between physical activity and sleep quality. Psychological resilience is defined in terms of a process-theoretic understanding of psychological resilience as the process by which individuals gradually adapt and resolve dilemmas when faced with negative life events (Lian, 2022). Studies have shown that psychological resilience and sleep quality are significantly negatively correlated (Sun, 2018) and that psychological resilience is a significant negative predictor of sleep quality (Lian, 2022). A study by Jianwen et al. (2020) on the relationship between psychological resilience and sleep quality found that high school students with high levels of psychological resilience had better sleep quality and were less likely to suffer from insomnia. Yukun et al. (2019) showed that psychological resilience was significantly associated with sleep quality and that improving psychological resilience could reduce the direct and indirect effects of stressful life events on sleep quality. Thus, psychological resilience may have an important predictive role for sleep quality.

In addition, Huiru et al. (2022) examined the relationship between physical activity and psychological resilience in adolescents following the 2019 coronavirus disease (COVID-19) pandemic and showed that there was a significant positive correlation between physical activity and psychological resilience, with physical activity being a significant positive predictor of psychological resilience. Siyu et al. (2021) showed that physical activity levels of college students were significantly associated with psychological resilience, and that there were significant differences in psychological resilience between college students with different levels of physical activity. Li et al. (2022) concluded that there was a significant positive correlation between physical activity and psychological resilience in college students to varying degrees, and that psychological resilience in college students gradually increased with their level of physical activity. Other studies have also shown that physical activity plays an important role in college students’ resilience, that physical activity is positively related to resilience, and that physical activity can improve resilience (Shanshan et al., 2021).

Thus, individuals with higher levels of physical activity may have higher levels of psychological resilience. In summary, physical activity may be closely related to psychological resilience and may further influence sleep quality through psychological resilience. Thus, we propose the following hypotheses: H2. Physical activity positively predicts psychological resilience and psychological resilience negatively predicts sleep quality, and that physical activity predicts sleep quality in college students through the mediation of psychological resilience.

Social adaptation may also play an important role in the relationship between physical activity and sleep quality. Social adaptation is the process by which individuals socialize by changing themselves or conforming to their environment (Tan et al., 2018). The results of the study show that there are many factors affecting the sleep quality of college students, such as stress, environmental factors, physiological changes, and personality traits, among which personality traits are in turn closely related to social adaptation, which is negatively correlated with sleep quality, and sleep disorders and social adaptation influence each other and interact with each other (Yuan et al., 2015). According to research, social adaptation affects not only the physical and mental health of college students, but also the quality of their sleep (Yan and Zhu, 2013). Many of the psychological problems of college students stem from maladjustment, and the negative emotions generated by maladjustment can easily lead to sleep disorders, and sleep disorders and social adaptation are mutually influential and interactive (Yuan et al., 2015). Through a study on sleep and adjustment of 2,166 freshmen, Liu (2019) pointed out that campus adjustment and satisfaction under the social adaptation dimension could significantly predict the sleep quality of university freshmen. Therefore, social adaptation may have a significant predictive role for sleep quality.

For many years, scholars have generally agreed that physical activity is one of the effective interventions for the development of positive psychological traits and harmonious physical and mental development in young people. Zhu and Shu (2022) used a cross-lagged research design to explore the causal relationship between physical activity and social adaptation among adolescents. The results indicated that physical activity was a causal variable for social adaptation, that means urging and encouraging adolescents to be physically active was effective in enhancing their social and coping social adaptation. The empirical study shows that physical activity level can significantly and positively predict the social adaptation of college students, and the higher the physical activity level of college students, the stronger their social adaptation ability (Li, 2021). Yang et al. (2013) suggested that physical activity has a more significant predictive effect on secondary school students’ mental health and is an effective means for adolescents to develop social adaptation. Physical activity overall contributed to the positive development of adolescents’ social adaptation, and contributed slightly more to positive adjustment than to the improvement of maladjustment. Active use of free time and long-term, high-frequency participation in group ball games and outdoor physical exercise in an open environment can maximize the effect of physical activity on the development of young people’s social adaptation, but blindly increasing the intensity of exercise has the opposite effect (Tan and Li, 2022). Another study showed that the social adaptation of college students who exercised more than five times a week was significantly higher than those who never exercised, but there was no significant difference between those who participated one to two times a week and those who participated more than three times a week but for less than 30 min each time, and too much exercise did not lead to more benefits (Ma, 2017). Thus, physical activity may be an important predictor of social adaptation.

In summary, it is hypothesized that social adaptation mediates the effect of physical activity on sleep quality. We propose the following hypotheses: H3. Physical activity positively predicts social adaptation, while social adaptation negatively predicts sleep quality, and physical activity mediates the role of social adaptation in predicting sleep quality in college students.

Research has shown that there is a strong correlation between an individual’s level of psychological resilience and adaptability, with the higher an individual’s level of psychological resilience, the more effectively they can use resources to cope with difficult situations and the more control they have over their external environment (Rutter, 2000). Psychological resilience has a significant positive predictive effect on the adjustment of mobile children, with higher levels of psychological resilience being associated with better levels of self-confidence and emotional regulation (Wang and Lin, 2018). Research on general college students has found that the factors influencing individual adjustment include both internal and external environmental factors, with psychological resilience being one of the internal influences. A study by Yan et al. (2021) found that the psychological resilience of college students with hearing impairment was significantly and positively related to their adjustment, and that psychological resilience positively predicted the adjustment level of college students with hearing impairment. Liu et al. (2013) found that psychological resilience of adults with hearing impairment was significantly and positively related to their social support, and that psychological resilience and social support positively predicted the level of social adaptation of adults with hearing impairment. Yuan’s (2019) study concluded that the psychological resilience of senior nursing students was significantly and positively correlated with their social adaptation ability, and that psychological resilience was an important factor influencing their social adaptation. Liao et al. (2019) showed that there was a positive correlation between psychological resilience and psychosocial adaptation in patients with permanent intestinal stoma, and that increasing the level of psychological resilience in patients could promote adaptive behavior toward the stoma. Other studies have shown that there is a strong relationship between the psychological resilience and social adaptation of left-behind junior high school students, and that psychological resilience positively predicts social adaptation; the higher their psychological resilience, the better their ability to adapt socially (Xie and Zou, 2015). Based on this, we propose the following hypotheses: H4. Psychological resilience and social adaptation play a role in mediating the chain between physical activity and sleep quality.

**FIGURE 1:**
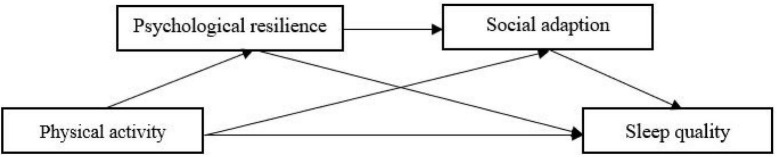
Hypothetical model.

In summary, this study had four main aims: (1) to examine the positive predictive role of physical activity on sleep quality in college students; (2) to examine the mediating role of psychological resilience between physical activity and sleep quality in college students; (3) to examine the mediating role of social adaptation between physical activity and sleep quality in college students; (4) to examine the chain of psychological resilience and social adaptation between physical activity and sleep quality mediating role (as shown in Figure 1).

At the same time, this study is of great significance to clinicians, patients, and researchers. It enables them to find out the sleep problems of patients in time and provide suggestions for patients to improve their sleep quality.

## Materials and methods

### Procedure and participants

**FIGURE 2:**
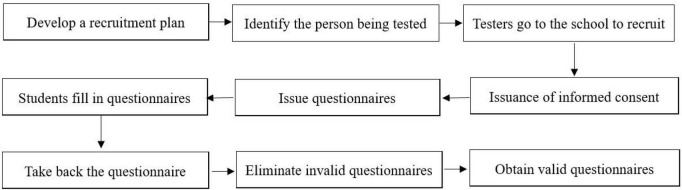
Recruiting flowchart.

From August to September 2022, a cross-sectional survey was conducted by using the convenience sampling method in Guangdong Province. Participants came from Jinan University, South China Normal University, Zhaoqing University, and Guangzhou Sport University, invite them to fill in the *Physical Activity Scale*, *Pittsburgh Sleep Quality Index Scale* (PSQI), *Psychological Resilience Scale*, and *Social Adaptation Diagnostic Scale* to collect data. The sample size is based on the rule of thumb, and all participants were informed and agreed to participate in the study. The main test was conducted by researchers majoring in psychology. Participants filled out questionnaires in the classroom and collected them on the spot. The answer time of each questionnaire was 5–15 min. A total of 1,689 students participated in the survey and 1,622 questionnaires were returned, with a valid recovery rate of 96.03%. The age range of the subjects was 18–23 years old, with an average age of 21.18 ± 1.518, of which 893 were male and 729 were female. A total of 395 were freshmen, 460 were sophomores, 398 were juniors, and 369 were seniors. The recruitment flow chart is shown in Figure 2.

The study has been approved by the Ethics Committee of Zhaoqing University and all processes follow the Helsinki Declaration.

### Measures and instruments

#### Physical activity

The *Physical Activity Rating Scale* (PARS-3) compiled by Liang (1994) was used to measure physical activity. The scale includes three items. The amount of physical activity was investigated from three aspects: physical activity intensity, physical activity time, and physical activity frequency. The level of physical activity participation was measured by the amount of physical activity. Each aspect was divided into five grades and scored according to 1–5 points, total score of physical activity = intensity of physical activity × (physical activity time–1) × physical activity frequency. The range is 0–100 points. The higher the score, the higher the individual’s physical activity. The study proved that the scale has good reliability in the Chinese junior high school student population (Feng et al., 2015). In the present study, the *Cronbach’s α* for this scale was 0.732.

#### Psychological resilience

Psychological resilience was assessed using the *Brief Resilience Scale* (BRS) revised by Chen et al. (2020), which is a unidimensional scale with six items on a five-point Likert scale ranging from “1 = totally disagree” to “5 = totally agree.” There are three positive and three negative scoring items each. The higher the score, the higher the psychological resilience. The scale has shown high reliability and validity among Chinese college students (Zhou and Zhou, 2022). In this study, the *Cronbach’s α* for this scale was 0.713.

#### Social adaptation

The scale of college students’ social adaptation questionnaire compiled by Fang (2008) is composed of 23 questions and is divided into five dimensions: learning adaptation, interpersonal adaptation, environmental adaptation, psychological adaptation, and future adaptation. The Likert five-point scoring method is adopted, from “1 = completely inconsistent” to “5 = completely consistent.” Calculate the average score of each item. The higher the score, the higher the social adaptation level. The scale shows high reliability and validity in Chinese college students (Tan et al., 2018). In this study, the *Cronbach’s α* for this scale was 0.954.

#### Pittsburgh Sleep Quality Index Scale

The PSQI developed by Buysse et al. (1989) and revised by Liu and Tang (1996) was used to measure the sleep quality of college students in the past month. A total of 18 questions were used to investigate the sleep quality of adolescents from seven dimensions: sleep quality, time to sleep, sleep time, sleep efficiency, sleep disorders, hypnotics, and daytime dysfunction. Each dimension was scored by five points, and 0–3 points were calculated according to the quality of sleep. The total score was obtained for all items. The higher the score, the worse the sleep quality. The scale shows high reliability and validity in Chinese college students (Liu et al., 2022). In this study, the *Cronbach’s α* for this scale was 0.914.

#### Statistical analyses

After data collection is complete, import the data to the SPSS 23.0 software. Firstly, delete the regular, incomplete, and missing questionnaires. Secondly, examine the internal consistency coefficients of the research instruments. Thirdly, conduct common method bias test on the collected data. Fourthly, use *PEARSON* correlation analysis to the main variables of the study. Fifthly, use Model 4 in the PROCESS plug-in to test the mediation effect of psychological resilience and social adaptation. Lastly, use Model 6 in the PROCESS plug-in to test the chain mediation effect of psychological resilience and social adaptation.

## Results

### Common method bias test

To test the consistency of the data, the Harman Single Factor Test was used to test for common method bias in this study. The results showed that the variance explained by the first factor was 26.457%, less than the critical value of 40%. This shows that there is no common method bias in this study.

### Descriptive statistics and correlation analysis of variables

As shown in Table 1, the correlation coefficients of physical activity, sleep quality, psychological resilience, and social adaptation are statistically significant. Correlation analysis showed that physical activity was positively correlated with psychological resilience and social adaptation, and negatively correlated with sleep quality; there was a significant negative correlation between sleep quality and psychological resilience and social adaptation; there is a significant positive correlation between psychological resilience and social adaptation. The relationship between variables supports subsequent hypothesis testing.

**TABLE 1:** Means, standard deviations, and correlations among variables.

Variable	*M*	SD	Gender	1	2	3	4
Gender			1				
1. PA	48.64	24.918	-0.007	1			
2. SQ	7.48	2.209	-0.067**	-0.237**	1		
3. PR	16.5	4.298	0.019	0.215**	-0.571**	1	
4. SA	89.27	20.270	-0.002	0.273**	-0.609**	0.539**	1

*N* = 1,622; PA, physical activity; SQ, sleep quality; PR, psychological resilience; SA, social adaptation. **p < 0.01.

### Demographic characteristics of the study sample

As shown in Table 2, 55.06% (893 people) of the total sample are male and 44.94% (729 people) are female. The level of psychological resilience of boys was significantly higher than that of girls. There was no significant difference in physical activity, sleep quality, and social adaptation between boys and girls.

**TABLE 2:** Differences in gender.

Variable	Gender	Number [*N* (%)]	*M*	SD	*t*	*p*
PA	Male	893 (55.06)	48.8	25.358	0.286	0.775
	Female	729 (44.94)	48.45	24.384		
PR	Male	893 (55.06)	7.61	2.206	2.684	0.007
	Female	729 (44.94)	7.31	2.202		
SA	Male	893 (55.06)	16.43	4.705	-0.745	0.456
	Female	729 (44.94)	16.59	3.741		
SQ	Male	893 (55.06)	89.31	19.524	0.096	0.923
	Female	729 (44.94)	89.21	21.162		

*N* = 1,622; PA, physical activity; SQ, sleep quality; PR, psychological resilience; SA, social adaptation.

### The mediation effect test between psychological resilience and self esteem

According to Ye and Wen’s (2013) suggestions on the test of intermediary effect, the model 6 in SPSS macro degree compiled by Hayes was used to complete the test of intermediary effect. The inspection results are shown in Table 3. First, controlling for demographic variables (gender and age), the direct pathway of physical activity on sleep quality was examined. The results showed that the model fitted well: *χ^2^/df* = 2.576, NFI = 0.935, GFI = 0.928, RMESA = 0.032, CFI = 0.904, and TLI = 0.951. The direct pathway of physical activity on sleep quality was significant before the inclusion of mediating variables (β = −0.2355, *t* = −9.8875, *p* < 0.01). Therefore, hypothesis 1 holds.

**TABLE 3:** Analysis of regression relationship among variables.

Effect	Item	Effect	SE	*t*	*p*	LLCI	ULCI
Direct effect	PA ⇒ SQ	-0.0531	0.0188	-2.8252	0.0048	-0.0900	-0.0162
Indirect effect	PA ⇒ PR	0.2145	0.0243	8.8225	0.0000	0.1668	0.2621
	PA ⇒ SA	0.1635	0.0210	7.7730	0.0000	0.1222	0.2048
	PR ⇒ SA	0.5036	0.0210	23.9614	0.0000	0.4624	0.5449
	PR ⇒ SQ	-0.3374	0.0215	-15.7106	0.0000	-0.3795	-0.2952
	SA ⇒ SQ	-0.4051	0.0218	-18.5579	0.0000	-0.4479	-0.3623
Total effect	PA ⇒ SQ	-0.2355	0.0238	-9.8875	0.0000	-0.2822	-0.1887

N = 1,622; PA, physical activity; SQ, sleep quality; PR, psychological resilience; SA, social adaptation.

The role of psychological resilience and social adaptation in mediating the chain between the relationship between physical activity and sleep quality was then examined. The results showed that *χ^2^/df* = 2.630, NFI = 0.946, GFI = 0.913, RMESA = 0.050, CFI = 0.924, and TLI = 0.915, indicating a good model fit. As shown in Table 3, analysis of the main variable relationships indicated that the direct effect of physical activity on sleep quality remained significant after the inclusion of mediating variables (β = −0.0531, *t* = −2.8252, *p* < 0.01) and the remaining direct pathways all reached significant levels and could act on sleep quality through three indirect pathways: (1) physical activity → psychological resilience → sleep quality; (2) physical activity → social adaptation → sleep quality; (3) physical activity → psychological resilience → social adaptation → sleep quality. Hypothesis 2 was tested by the significant direct paths of physical activity on psychological resilience (β = 0.2145, *t* = 8.8225, *p* < 0.01) and psychological resilience on sleep quality (β = −0.3374, *t* = −15.7106, *p* < 0.01); the significant direct paths of physical activity on social adaptation (β = 0.1635, *t* = 7.7730, *p* < 0.01) and the direct pathway of social adaptation to sleep quality was significant (β = −0.4051, *t* = −18.5579, *p* < 0.01), hypothesis 3 was tested; the direct pathway of psychological resilience to social adaptation was significant (β = 0.5036, *t* = 23.9614, *p* < 0.01), indicating that a chain mediator of psychological resilience and social adaptation existed and hypothesis 4 was tested.

**FIGURE 3:**
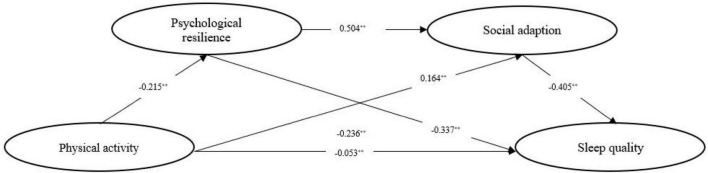
Chain mediation model. **p < 0.01.

The deviation correction percentile Bootstrap method (repeated sampling 5,000 times) was used for inspection. It can be seen from Table 4 that the 95% confidence interval result of the intermediary effect Bootstrap: the confidence interval of physical activity → psychological resilience → sleep quality (−0.0977, −0.0503), and the intermediary effect amount is −0.0723; confidence interval of physical activity → social adaptation → sleep quality (−0.0929, −0.0453), and intermediary effect amount −0.0662; The confidence interval of physical activity → psychological resilience → social adaptation → sleep quality (−0.0613, −0.0296), the amount of intermediary effect is −0.0438, and the confidence interval does not include 0, indicating that the intermediary effect is significant. The mediating effect of psychological resilience and social adaptation between physical activity and sleep quality is shown in Figure 3.

**TABLE 4:** Mediating effect and effect size.

Path	Effect	The proportion of mediations in the total effect	95% Confidence interval	
			**Lower limit**	**Upper limit**
PA→PR→SQ	−0.0723	−0.0723/−0.2355 = 30.70%	−0.09770	−0.0503
PA→SA→SQ	−0.0662	−0.0662/−0.2355 = 28.11%	−0.0929	−0.0453
PA→PR→SA→SQ	−0.0438	−0.0438/−0.2355 = 18.60%	−0.0613	−0.0296

N = 1,622; PA, physical activity; SQ, sleep quality; PR, psychological resilience; SA, social adaptation.

## Discussion

The results show that the level of psychological resilience of boys was significantly higher than that of girls and there was no significant difference in physical activity, sleep quality, and social adaptation between boys and girls. At the same time, physical activity, sleep quality, psychological resilience and social adaptation are statistically significant, and physical activity can not only directly and significantly predict sleep quality, but also predict sleep quality through the chain mediation of psychological resilience and social adaptation. The relationship between physical activity and sleep quality of college students was further explained.

### Physical activity and sleep quality

This study shows that physical activity among college students can effectively predict sleep quality, which is consistent with previous relevant research results and verifies hypothesis 1 (Pedrosa et al., 2022). Merellano et al. (2022) concluded that being male and having a high level of physical activity during isolation is conducive to good sleep quality, regardless of age. Yao et al. (2022) believed that there was a significant correlation between physical activity and sleep. For those with sleep problems, it is recommended to increase moderate intensity physical activity to moderate and high levels. Previous research has shown that physical activity promotes mental health by secreting hormones such as dopamine (Archer et al., 2014). Reducing negative emotions can improve sleep quality (Zhao et al., 2020). The study also found that scientific and effective adherence to physical activity can reduce the level of serum cortisol and improve the body’s immunity, while the continuous improvement of sleep quality is closely related to the lower level of serum cortisol and immunity (Deng et al., 2018). Yang (2020) also pointed out that through aerobic training and aerobic combined resistance training, two kinds of exercise interventions can inhibit excessive arousal, regulate anxiety, tension and other negative emotions, and thus improve the quality of sleep. The study also found that resistance training can promote the release of IL-6 from skeletal muscle for immune regulation, which can improve the quality of sleep to a certain extent. However, some researchers have proposed that the level of physical activity will have a negative impact on sleep quality, such as physical activity with too high intensity or too late. Therefore, when increasing the amount of physical activity is used to improve sleep quality, attention should still be paid to selecting the time and intensity of physical activity.

### Independent mediating effect of psychological resilience

This study found that psychological resilience played a mediating role between physical activity and sleep quality, which verified hypothesis 2. This is consistent with previous relevant research evidence, that is, physical activity significantly positively predicts psychological resilience and psychological resilience significantly negatively predicts sleep quality (Xu, 2018; Zhang et al., 2021). This study included three variables at the same time, revealing that physical activity is an important factor in promoting psychological resilience and improving sleep quality.

On the one hand, psychological resilience has a negative predictive effect on sleep quality. Psychological resilience is characterized by the ability to recover from stress, which is critical to sleep quality. According to the neuroscience theory of resilience, resilience plays a mediating role in the relationship between middle front gross (MFG) functional connectivity and sleep quality, providing new insights into the relationship between resilience and sleep quality (Yan et al., 2022). The neural mechanism of psychological resilience also believes that psychological resilience is related to brain regional structure, neural circuit, and brain neural network, and brain function plays an important role in emotional regulation, thus indirectly affecting sleep quality (Yang et al., 2019). The protective mechanism of psychological resilience believes that the better the individual’s psychological resilience is, the better he or she will be able to cope with the adversity in learning and life, reduce the impact of negative stress events on himself or herself, face problems in a positive way, and promote the development of physical and mental health. Therefore, as an important psychological resource, psychological resilience plays a protective role in sleep quality (Babak et al., 2021).

On the other hand, physical activity positively predicted psychological resilience. The entertaining, open and competitive characteristics of sports situation provide a better space for the development of college students’ psychological resilience. In the related research on the development of psychological resilience, physical exercise is regarded as an important influencing factor. Regular physical exercise can improve psychological resilience, including reducing physical and psychological responses to stress, improving individual physiological and psychological status, and acting as a buffer for stress (Belcher et al., 2020). The prevention model of psychological resilience development believes that every kind of pressure that is not too strong is a challenge to the individual, but it can also enhance psychological resilience after the pressure is overcome (Yang et al., 2019). In the process of physical activity, college students will encounter the pressure related to learning sports skills, challenges from competitors and the sense of frustration brought about by them. These may not be conducive to the mental health of college students in the short term. However, after successfully overcoming these problems through their own efforts or with the support of their peers and teachers, they can form a more flexible attitude and improve their ability to solve problems, then they can effectively cope with and adapt to the situation of passion in life, and maintain a positive attitude toward life and emotional state. Sports have diversified functions. Physical activity can not only improve the physical and mental health of college students, but also an important way to enhance their will quality (Pan and Yu, 2015). Through physical activity, students’ attention orientation can be adjusted, their cognitive function can be improved, and their emotional regulation level can be improved, which are all related to the internal forming factors of their psychological resilience (Liu, 2020). Therefore, physical activity can improve the development of sleep quality by enhancing students’ psychological resilience.

### Independent mediating effect of social adaptation

This study also found that social adaptation plays a mediating role between physical activity and sleep quality, which confirms hypothesis 3 of this study. This is consistent with previous relevant research evidence, that is, physical activity positively predicts social adaptation, and social adaptation further predicts sleep quality (Wang, 2011). This study included three variables at the same time, revealing that physical activity is an important factor to improve individual social adaptation, and also an important factor to improve sleep quality.

There is a significant negative correlation between college students’ social adaptation and sleep quality. Research shows that sleep quality is positively related to stress, negative coping, and negative emotions (Li, 2021). Individuals with higher social adaptability also have higher sleep quality. The possible reason is that college students with good social adaptability not only include the ability to adapt to practical work with the book knowledge they have learned, but also include the ability to coordinate interpersonal relationships and living environment harmoniously. Individuals with this ability can keep good contact with the natural environment and social environment, and have good adaptability to the surrounding environment. They have certain communication ability, can effectively cope with the pressure of daily life and work, and can work, study and live normally. When individuals face setbacks and failures or sudden stress events, they can take appropriate behavior without excessive stress reaction and have strong self-regulation ability. All these important psychological resources are important internal resources for overcoming sleep disorders and thus are more conducive to improving their sleep quality (Yuan et al., 2015).

The study confirmed that physical activity was a causal variable for adolescents’ social adaptation, i.e., adolescents’ physical activity significantly predicted later social adaptation, and the results were generally consistent with Sun et al. (2019). The theory of socially adaptive development suggests that human society develops based on continuous learning and exploration of life practices. It follows that physical activity, as a positive social interaction activity, can create an opportunity for young people to interact with each other and with society, enabling them to exchange emotions with their peers, build a wider interpersonal circle, and enhance their social adaptation skills (Chen and Yu, 2015). As a type of challenging social practice, physical exercise is often accompanied by difficulty, task challenge, self-improvement and goal attainment, enabling individuals to continuously experience learning, coping and self-representation in exercise practice, achieving a balance between themselves and the environment through assimilation and adaptation, and enhancing their ability to deal with and adapt to situations. Regular physical exercise not only strengthens the body and builds the personality, but also enables young people to stimulate their sense of teamwork, develop interpersonal communication skills and enrich their skills and techniques in handling and solving problems through the practice of physical exercise, so as to improve their overall social, handling and interpersonal relations and other social adaptation skills and reduce externalized problem behaviors (Bornstein et al., 2010). It is for this reason that we should urge and encourage young people to be physically and mentally active in order to enhance their social adaptation.

### The chain mediating effect of psychological resilience and social adaptation

This study found that college students with higher levels of psychological resilience had greater social adaptation. Adaptation is the interaction between an individual and his or her environment during the process of growth, and is manifested in dynamic changes in emotions, cognition and behavior, in which the individual constantly adjusts his or her adaptive state according to the environment. Psychological resilience is an important protective factor for mental health (Zhang and Tian, 2014). The higher the level of psychological resilience, the more psychological resources an individual has, the more he is able to mobilize and allocate his psychological resources, such as optimism and strength, according to the environment, in order to recover from negative experiences and adapt flexibly to the external environment. The Dynamic Model of Psychological resilience suggests that psychological resilience is an innate ability of individuals who need to meet their own needs for security, belonging, love and respect as they grow up, and that the material and spiritual support they receive from family, peers and society during physical activity is an important resource for meeting these needs. If these external protective factors meet the individual’s psychological needs, the individual will actively develop his or her own good traits, improve his or her psychological resilience and social adaptation, and thus improve the quality of his or her sleep. Therefore, the higher the level of physical activity of college students, the more social support they receive, i.e., the more care, support and assistance they receive from their parents, friends and teachers, etc., the higher their level of self-confidence, the lower their level of anxiety and depression, the better their psychological resilience, the better they are at mobilizing all kinds of resources to actively cope with setbacks, difficulties and environmental changes and other aspects of stress, promoting improved social adaptation and eliminating sleep. The higher the level of self-confidence, the better the college students will be at mobilizing resources to cope positively with frustration and stressful situations such as environmental change, promoting social adaptation, and eliminating sleep disorders.

### Practical significance

The present study examined the effects of physical activity on sleep quality, enriching related research in the field of physical activity and sleep quality with practical implications for improving sleep quality among college students. First, physical activity is an important predictor variable not only of sleep quality, but also of psychological resilience and social adaptation. According to current research evidence, sleep problems among college students are increasing, and physical activity can be an adjunct to non-pharmacological interventions to improve sleep quality. Therefore, physical activity should be encouraged and supported to develop good lifestyles and habits in order to improve the sleep problems of Chinese college students. It is recommended that college students pay attention to the selection of physical activity programs, intensity, time, and frequency, so that they can be persistent and challenging, and that they can improve their social adaptation and psychological resilience in the process of physical activity in order to prevent and reduce sleep quality problems more favorably. Secondly, psychological resilience and social adaptation are important factors influencing the quality of sleep of college students. The mediating role of psychological resilience and social adaptation suggests that university educators should pay attention to the impact of psychological resilience and social adaptation on the sleep quality of college students and use psychological resilience and social adaptation growth group counseling interventions to improve the level of psychological resilience and social adaptation of college students, to improve their ability to cope with stressful events, to be able to look at problems in an objective and rational manner, and to be proactive in adapting to life, school and work. This will enhance the effect of physical activity on the quality of sleep of college students and reduce their sleep quality problems.

### Limitations and prospects

Firstly, this study used a self-report questionnaire, and data could be collected in the future using a combination of others’ ratings and self-reports. Secondly, this study adopted a cross-sectional research method to explore the mechanisms of physical activity on sleep quality among college students, but this research method could not draw inferences about the causal relationships between variables, and future in-depth research could be conducted using a longitudinal follow-up or experimental intervention design. Future research could focus on different types of universities in different regions to further validate the findings of this study. Finally, this study only considered the mediating effect of psychological resilience and social adaptation between physical activity and sleep quality, while there may be other mediating variables such as perceived stress and social support that need to be investigated in depth.

## Conclusion

(1) Physical activity is a significant positive predictor of psychological resilience and social adaptation and a negative predictor of sleep quality, suggesting that physical activity may help to improve psychological resilience and social adaptation and reduce sleep quality problems in college students. (2) Physical activity not only directly predicts sleep quality, but also indirectly predicts sleep quality through the separate mediation of psychological resilience and social adaptation, and indirectly predicts sleep quality through the chain mediation of psychological resilience and social adaptation. This suggests that in improving the sleep quality of college students, special attention should be paid not only to enhancing physical activity, but also to improving the psychological resilience and social adaptation of college students.

## Data availability statement

The original contributions presented in this study are included in the article/supplementary material, further inquiries can be directed to the corresponding author.

## Ethics statement

The study was approved by the Research Ethics Committee of Zhaoqing University (No. 2022-1110-01), and all subjects were informed of the purpose and characteristics of the study and signed an informed consent form.

## Author contributions

KG designed the study. YL collected and analyzed the data, wrote the manuscript, and revised the manuscript. Both authors contributed to the article and approved the submitted version.
